# Primary Prevention for Intermediate Risk: The More Things Change, the More They Stay the Same∗

**DOI:** 10.1016/j.jacadv.2024.100884

**Published:** 2024-03-05

**Authors:** Philip Greenland, Peter A. Glynn

**Affiliations:** a Department of Preventive Medicine, Northwestern University Feinberg School of Medicine, Chicago, Illinois, USA; b Department of Medicine (Cardiology), Northwestern University Feinberg School of Medicine, Chicago, Illinois, USA

**Keywords:** coronary calcium, primary prevention, cost-effectiveness analysis

**Figure:**
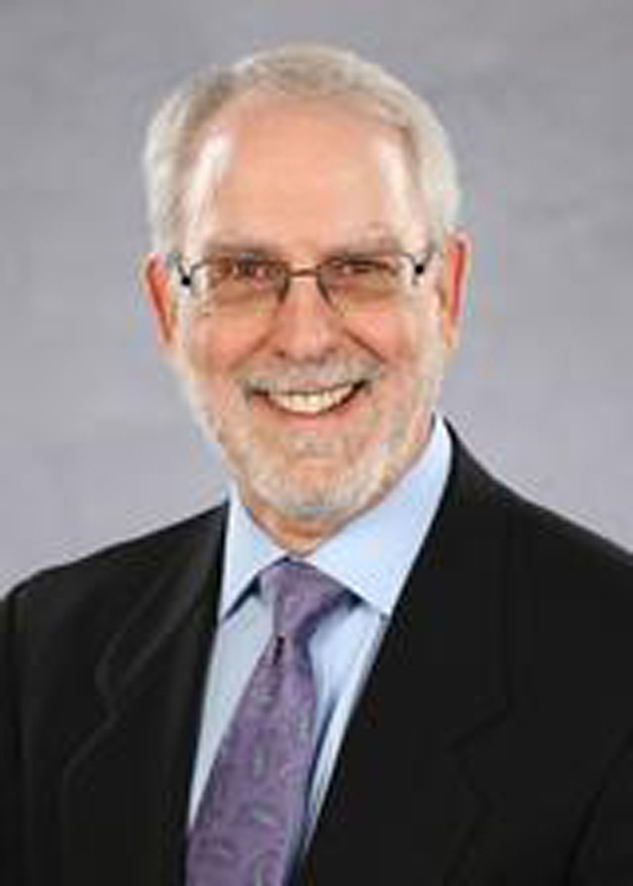

Coronary artery calcium (CAC) scoring is a potent tool in atherosclerotic cardiovascular disease (ASCVD) risk stratification. The presence and amount of CAC are directly associated with incident coronary heart disease event rates in middle-aged and older adults without known ASCVD.^1^ In asymptomatic people, a CAC score of zero portends very low coronary heart disease event rates as far out as 15 years.^2^ CAC scoring is recommended by the ACC/AHA primary prevention guidelines as a consideration in individuals at borderline (10-year ASCVD risk 5.0%-<7.5%) and intermediate risk (≥7.5%-20%) when the decision regarding statin therapy is uncertain.^3^ Because approximately 25% of the U.S. adults are estimated to be at intermediate ASCVD risk^4^ and because of burgeoning cardiovascular care costs, it is essential to understand the economic impact of a CAC-driven approach to primary prevention.

While several studies have addressed this impact in the U.S. health care system, in this issue of *JACC: Advances*, Qureshi et al^5^ provide important new data on the cost-effectiveness of a CAC-guided treatment approach within the Canadian publicly funded health care system. The Canadian lipid guidelines,^6^ similar to the ACC/AHA guidelines,^3^ recommend statin initiation in intermediate-risk individuals with an elevated low-density lipoprotein cholesterol or other risk factors present. As such, the authors compared 2 strategies: universal statin treatment with a high-intensity statin in all intermediate-risk ASCVD-free individuals vs a CAC-guided approach, which featured 1-time CAC scoring and subsequent high-intensity statin therapy for those with CAC ≥ 1 and no therapy for those with CAC = 0. The authors used a state-transition microsimulation model to estimate costs and outcomes at 5 and 10 years among a hypothetical 10,000-person sample that mirrored the age and CAC distributions observed in the MESA (Multi-Ethnic Study of Atherosclerosis).^7^ Event rates were estimated using MESA data and adjusted for statin therapy use. Costs were estimated using Alberta administrative health datasets and published literature. Statin-related disutility and adverse events were accounted for, as were incidental findings and subsequent costs related to CAC scoring. The outcome of interest was the incremental cost-effectiveness ratio (ICER), calculated as the difference in health care costs divided by the difference in quality-adjusted life years (QALYs) associated with each approach. An ICER of CA<$50,000/QALY was set as the threshold for cost-effectiveness.

The authors found that a CAC-driven treatment approach was estimated to increase mean costs by CA$326 (95% CI: $325-$326) and CA$172 (95% CI: $169-$175) at 5 and 10 years respectively. By limiting statin-related disutility and statin-related adverse effects, the CAC-driven approach was associated with an increase in mean QALYs by 0.01 (95% CI: 0.01-0.01) and 0.02 (95% CI: 0.02-0.02) at 5 and 10 years. This corresponded to an ICER of CA$54,492 (95% CI: $52,342-$56,816) and CA$8,118 (95% CI: $7,968-$8,279) at 5 and 10 years. The authors concluded that a CAC-driven approach, in comparison to universal statin therapy, was essentially cost-neutral at 5 years and cost-effective at 10 years among intermediate-risk, ASCVD-free, statin-eligible adults in Canada. The model was sensitive to statin cost, CAC cost, and disutility related to statin therapy, with high statin costs, low CAC costs, and high statin disutility all favoring a CAC-driven approach.

These findings are generally consistent with the findings of previous cost-effectiveness analyses carried out in the U.S. population, reviewed in Hong et al.^8^ These analyses make slightly different assumptions but are generally similar in approach. With 1 exception, they all found that CAC was cost-effective or identified circumstances in which CAC was cost-effective. Much as in the present study, the sensitivity analyses found a CAC-driven approach to be cost-effective when statin costs are high, CAC costs are low, and there is disutility associated with statin use.^8^

A key point of both Hong et al^8^ and the present study is that while a CAC-driven approach is cost-effective, the clinical and economic outcomes of CAC-driven approach and a universal treatment approach are remarkably similar. This knowledge allows for a flexible, patient-centric approach and also helps accommodate the variability in local practice environments intrinsic to such large and diverse markets as the United States and Canada. Where CAC costs are low and statins may be more expensive, then a CAC-driven strategy may predominate. On the contrary, in locations where CAC is not available or where its costs are high, a universal statin approach could predominate. The current guidelines allow for such flexibility.

Society guidelines, the United States Preventive Task Force,^9^ and real-world data^10^ all support the use of statins in primary prevention for intermediate-risk patients. Unfortunately, guidelines are not always reflected in reality. Recent data from the U.S. National Health and Nutrition Survey from 2011 to 2018 suggests that nearly 80% of patients at borderline and intermediate ASCVD risk are not taking statin currently, despite over 50% of patients in both groups having at least 1 additional risk-enhancing factor.^11^ No reasonable cost-effectiveness analysis would assume that 22% of intermediate risk patients are prescribed statins; of course, they would not perform as well in terms of cost or outcomes. However, that is our unfortunate reality, particularly for Black patients, women, and individuals living in areas of poverty.^12^

How can we reverse this predicament? First, thoughtful clinician-patient discussions to combat the effects of misinformation regarding statins are essential. Second, novel systems-based approaches utilizing implementation science to improve statin prescription and adherence in primary prevention are desperately needed. These interventions must pay special attention to closing the disparity gap. Finally, these approaches must be borne out in trials proving their effectiveness in real-world health systems. Until then, despite the accumulation of evidence supporting CAC, the proliferation of novel lipid lowering agents, or the arrival of novel cardiometabolic agents, we will sadly find that in primary prevention, the more things change, the more they stay the same.

## Funding support and author disclosures

Dr Greenland has been supported by the 10.13039/100000002National Institutes of Health and the 10.13039/100000002American Heart Association. Dr Glynn has reported that he has no relationships relevant to the contents of this paper to disclose.
